# Global prevalence of chronic fatigue syndrome among long COVID-19 patients: A systematic review and meta-analysis

**DOI:** 10.1186/s13030-022-00250-5

**Published:** 2022-10-23

**Authors:** Nader Salari, Yassaman Khodayari, Amin Hosseinian-Far, Hosna Zarei, Shabnam Rasoulpoor, Hakimeh Akbari, Masoud Mohammadi

**Affiliations:** 1 Department of Biostatistics, School of Health, Kermanshah University of Medical Sciences, Kermanshah, Iran; 2 Student Research Committee, Kermanshah University of Medical Sciences, Kermanshah, Iran; 3 Department of Business Systems & Operations, University of Northampton, Northampton, UK; 4 Department of Psychiatric Nursing, School of Nursing and Midwifery, Urmia University of Medical Sciences, Urmia, Iran; 5 Cellular and Molecular Research Center, Gerash University of Medical Sciences, Gerash, Iran

**Keywords:** Prevalence, Fatigue, Chronic fatigue syndrome, COVID-19, Coronavirus, Meta-analysis, Systematic review

## Abstract

**Background:**

Chronic fatigue syndrome is a persistent and debilitating disorder. According to several studies, chronic fatigue syndrome has been identified among recovered COVID-19 patients as the most common symptom of long COVID. The aim of this systematic review and meta-analysis study was to obtain the prevalence of chronic fatigue syndrome in long COVID cases.

**Methods:**

In this systematic review and meta-analysis, we analysed reported results of studies that assessed the occurrence of chronic fatigue syndrome among COVID-19 patients four weeks after the onset of symptoms. The study selection was commenced by searching PubMed, Web of Science, Science Direct, Scopus, Embase, and Google scholar using the keywords of Chronic fatigue syndrome, COVID-19, and post-COVID-19 syndrome. The searches were without a lower time limit and until April 2022. Heterogeneity of studies was assessed using the I^2^ index, and a random effects model was used for analysis. Data analysis was performed within the Comprehensive Meta-Analysis software (version 2).

**Results:**

The pooled prevalence of chronic fatigue syndrome four weeks after the onset of COVID-19 symptoms, in 52 studies with a sample size of 127,117, was 45.2% (95% CI: 34.1-56.9%). Meta-regression analysis in examining the effects of the two factors of sample size, and year of study on the changes in the overall prevalence, showed that with increasing sample size, and year of study, the prevalence of chronic fatigue syndrome among long COVID patients (p < 0.05).

**Conclusion:**

Our results show that the overall prevalence of chronic fatigue syndrome as a long COVID symptom is 45.2%. Chronic fatigue after infection with COVID-19 can negatively affect personal and social lives. Given such significant negative consequences caused by the syndrome, it is recommended that health policymakers allocate funds to reduce the adverse effects of this syndrome, by creating programs to support long COVID patients.

## Introduction

The novel Coronavirus 2019 (COVID-19) is a highly contagious respiratory disease caused by the severe acute respiratory syndrome coronavirus 2 (SARSr-CoV-2), and its spread was declared a pandemic by the World Health Organization (WHO) in March 2020 [1]. Since the disease inception in late 2019, there has been a major clinical emphasis on respiratory manifestation among patients [2]. The clinical symptoms of the disease among COVID-19 patients are very different [2, 3]. While some people are asymptomatic, a number of patients experience symptoms that are generally similar to other viral respiratory illnesses, such as fever, cough, shortness of breath, headache, and sore throat [4, 5]. In the acute phase of COVID-19, several complications have reported, including negative effects of the disease on patients’ gastrointestinal tract, renal system, liver, and rheumatological and neurological systems [6, 7].

Long COVID (post-COVID-19 syndrome) was first introduced in early 2020 as part of the assessment of long-term and persistent COVID-19 symptoms [8]. Immediately after the development of the first cases of COVID-19, scientists observed that COVID-19 patients had symptoms that lasted for several weeks after acute infection. The most common symptoms remaining after recovery from the infection include fatigue, shortness of breath, olfactory and taste disorders, chest pain, myalgia, sleep and mental disorders; Symptoms may last for several months and disrupt the work activities and quality of life of affected individuals [9–12].

Fatigue is known as one of the important symptoms of post-COVID-19 syndrome and in some studies, the prevalence of fatigue has been reported up to 90% in patients in the acute phase of COVID-19 disease [13]. When fatigue cannot be justified with a medical condition, it is referred to as chronic fatigue syndrome [14]. Chronic fatigue syndrome is a long-term disease characterized by at least 6 months of fatigue and burnout [15]. Moreover, in an international online survey, 77.9% of patients with COVID 19 reported chronic fatigue syndrome 7 months after the onset of the disease [16].

Chronic fatigue syndrome (CFS) is characterized by profound tiredness, regardless of bed rest. Another name for it is myalgic encephalomyelitis/chronic fatigue syndrome (ME/CFS). Its symptoms may worsen with physical or mental activity. CFS can happen suddenly and last for years. The following are the most common symptoms of CFS. However, each person may experience symptoms differently. Symptoms may include Sensitivity to light, Headache, Tender lymph nodes, Fatigue and weakness, Muscle and joint pain, Inability to concentrate, Insomnia, Forgetfulness, and Depression. The symptoms of CFS may look like other medical conditions. Always talk with your healthcare provider for a diagnosis [16–20]. Myalgic encephalomyelitis/chronic fatigue syndrome (ME/CFS) is a complex, controversial, and common disease [14, 17–20].

People with chronic fatigue syndrome, like patients with other chronic illnesses, have significantly impaired functioning. More than 50% of patients require specialized care services that lead to significant personal, emotional, physical, and economic complications. Problems include the reporting of symptoms such as dizziness and tachycardia, which are expressed by the severity of fatigue among the patients [16, 21, 22].

Considering the COVID-19 pandemic and the increase of studies in this field and considering that many studies have been conducted in the field of chronic fatigue syndrome among long-term COVID-19 patients around the world, the heterogeneity of studies in this field is very high and there is still no study that It has not been done to investigate the overall chronic fatigue syndrome among long-term COVID-19 patients. This study aims to provide the pooled global prevalence of chronic fatigue syndrome among long COVID-19 patients through systematic review and meta-analysis.

## Methods

We conducted the initial search in February 2022. In this systematic review and meta-analysis and in order to find related studies, the PubMed, Web of Science, Science Direct, Scopus, Embase, and Google Scholar databases were searched using the keywords of Chronic fatigue syndrome, COVID-19, and post-COVID-19 syndrome. Meta-data of the identified articles were then transferred into the EndNote reference management software. To maintain the comprehensiveness of the search, the lists of references within the identified articles were manually reviewed. Searches were last updated in April 2022.

## Inclusion and exclusion criteria

Inclusion criteria were the conditions to include an article following the search process. The inclusion criteria used for study selection are:

- Studies that have reported the prevalence of chronic fatigue syndrome among long COVID patients.
- Studies in which the prevalence of fatigue has been reported at least four weeks after the onset of COVID-19 symptoms.
- Studies that their full text was available.
- Studies that provided sufficient data (sample size, and prevalence percentage).
- Observational studies (cross-sectional, control case, and cohort).

In contrary, exclusion criteria are used to reject or omit an article. These criteria are listed below:

- Case series.
- Review studies.
- Duplicate articles.
- Studies with insufficient data (failure to provide sample size, insufficient follow-up in terms of time from the onset of symptoms, and lack of prevalence reporting).

## Study selection

As highlighted earlier, EndNote was utilized to organize the list of identified articles. Initially, studies that were repeated in various databases were excluded from this systematic review, and only one copy was retained. In the initial screening phase, the titles and abstracts of the articles were carefully examined, and irrelevant articles were omitted. In the second stage, eligibility evaluation, the full texts of remaining articles were reviewed and studies that met the inclusion criteria were included in the review. To increase credibility and prevent bias, initial screening was performed by two reviewers (HZ and YKh), to determine whether the inclusion criteria were met. In cases of a disagreement between the two reviewers, the opinion of the third reviewer (SHR) was sought to reach a consensus. A total of 52 studies entered the third stage, i.e., quality evaluation.

## Quality evaluation

**Fig. 1:**
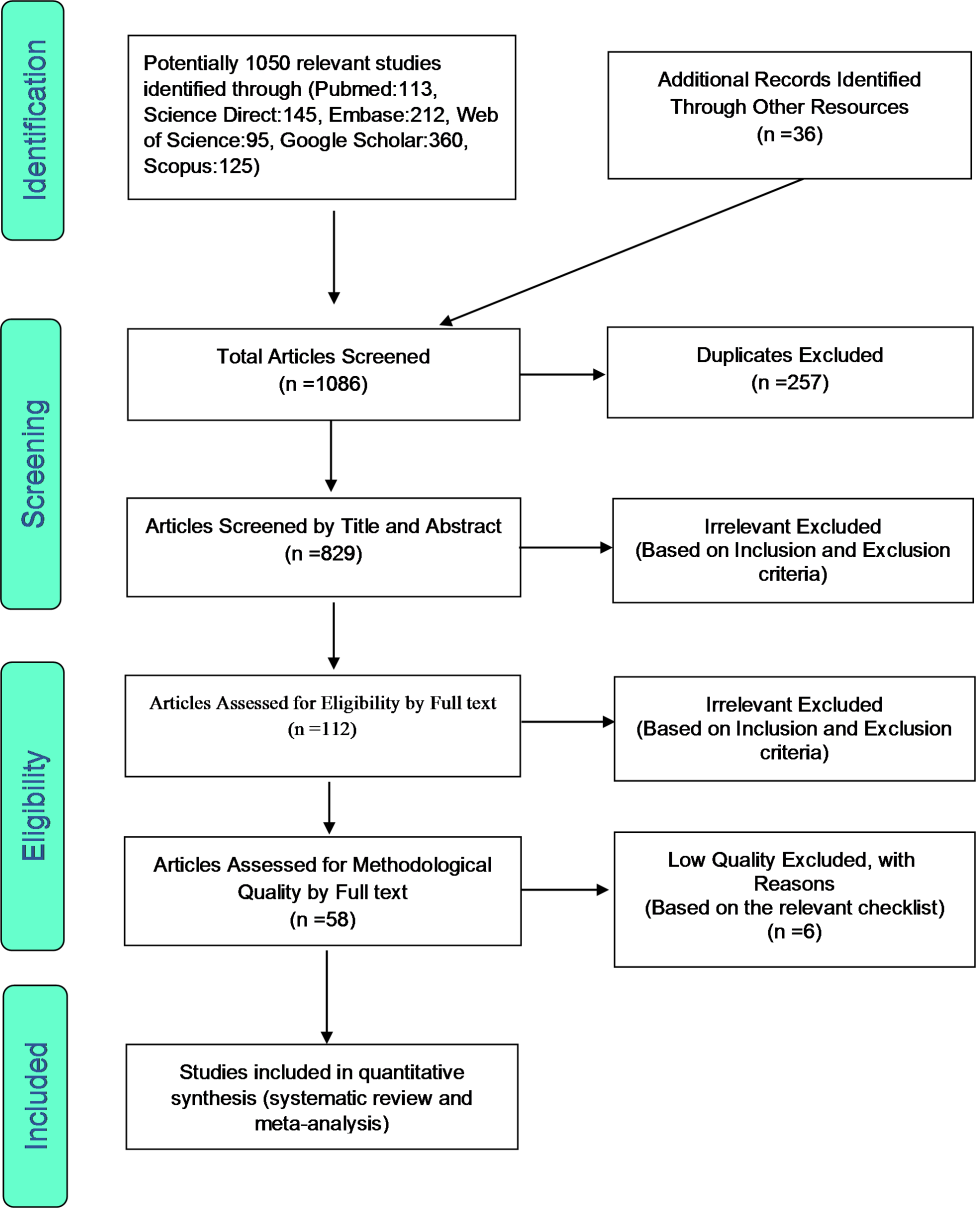
PRISMA flow diagram for study selection

In order to evaluate the quality of the remaining articles, a checklist appropriate to observational studies was used. Strengthening the Reporting of Observational studies in Epidemiology. (STROBE) consists of six scales including title, abstract, introduction, methods, results, and discussion. In total, these heading contains a total of 32 items/sub-headings. These 32 items include various methodological aspects of the study such as title, problem statement, study objectives, type of study, study statistical population, sampling method, sampling strategy, definition of variables and procedures, study data collection methods, statistical analysis methods and findings. If an article fulfils each of these 32 items, it scores 1 point. Accordingly, articles with scores of 16 and above were considered articles with medium and high quality respectively. Similarly, articles with scores below 16 were considered low in terms of methodological quality and were therefore excluded from the review. Finally, 52 articles were selected for review and meta-analysis. The PRISMA flow diagram (Fig.1) outlines the process.

### Data extraction

Data from the remaining articles were extracted by two researchers using a different pre-prepared checklist. This checklist included the following headings: First author’s name, year of publication, research location, sample size, gender, age, and prevalence of chronic fatigue syndrome (Table1).

### Statistical analysis

The I^2^ test was used to evaluate the heterogeneity of selected articles. To investigate the publication bias, and due to the high volume of samples included in the study, the Egger’s test was adopted at a significance level of 0.05, and the corresponding funnel plot was drawn. Data analysis was performed within the Comprehensive Meta-Analysis (CMA) software (version 2).

## Results

In this systematic review and meta-analysis of study, it was aimed to find out the global prevalence of chronic fatigue syndrome among long COVID patients. The systematic review searches were conducted without a lower time limit and until April 2022. The study selection and protocol for the review was in accordance with the Preferred Reporting Items for Systematic Reviews and Meta-Analyses (PRISMA) 2009 guidelines. Based on the initial search in the abovementioned databases, 1050 possible related articles were identified and transferred into the reference management software (EndNote), and an additional 36 articles were identified through a manually search of list of references. Out of a total of 1086 identified studies, 257 were duplicates, and were excluded. In the screening stage, out of 829 studies, 717 articles were removed, after the examination of titles and abstracts and based on inclusion and exclusion criteria. In the eligibility evaluation stage, out of the remaining 112 studies, 54 articles were omitted by reviewing the full text of the articles, due to lack of relevance, and based on inclusion and exclusion criteria. In the quality evaluation stage, by closely examining the full text of the remaining articles and by considering the scores obtained from the STROBE checklist, out of the remaining 58 studies, 6 studies with low methodological quality were excluded. Finally, 52 studies were included for the final analysis (Fig.1). Information of all included studies were extracted using a separate checklist and are presented in Table1.

**Table 1:** Characteristics of selected studies

Author	Y ear	Region	Study design	Age	Population	Men%	Prevalence%(N)
D.Bierle et al. [23]	2021	USA	Cross sectional	46.5	42	33.3%	74(31)
MAEG.Aly et al. [24]	2021	Egypt	Cross sectional	73.18	115	0%	57.4(66)
N. K. N. Anjana et al. [25]	2021	India	Cohort	41.5	154	37%	3.2(5)
A. Asadi et al. [1]	2021	Iran	Cross sectional	12	58	48%	21(12)
M. Agustin et al. [27]	2021	Germany	Cohort	43	353	42.7%	14.2(50)
M. Bell et al. [28]	2021	USA	Cohort	44	303	30%	37.5(113)
S. Bliddal et al. [29]	2021	Denmark	Cross sectional	N/A	445	42.9%	95(423)
B. Blomberg et al. [30]	2021	Norway	Cohort	46	272	49%	90(244)
B. Bouteleux et al. [31]	2021	France	Cross sectional	48	39	44%	77(30)
C. Brackel et al. [32]	2021	Netherlands	Cross sectional	13	89	N/A	87(77)
N. Chopra et al. [33]	2021	India	Cross sectional	34.9	53	53%	22.6(12)
B. cil et al. [34]	2022	Turkey	Cross sectional	50.25	396	50%	56.1(222)
N. Erol et al. [35]	2021	Turkey	Case control	9.16	121	53.71%	60(72)
R. Ganesh et al. [36]	2021	USA	Cross sectional	44	817	38.9%	90.6(739)
F. Gonzalez-Andrade et al. [37]	2021	USA	Cross sectional	39	1366	49.71%	67.3(919)
A. Iqbal et al. [38]	2021	Pakistan	Cross sectional	40.1	158	44.9%	82.9(131)
M. Islam et al. [39]	2021	Bangladesh	Cross sectional	34.7	1002	57.9%	11.5(115)
B. Kayaaslan et al. [40]	2021	Turkey	prospectiv	40.5	1007	54.4%	24.3(244)
C. Lemhofer et al. [41]	2021	Germany	Cross sectional	49.8	365	40.5%	37.5(137)
F.S. Mirfazeli et al. [42]	2021	Iran	Cross sectional	50	95	58%	23(22)
D. Munblit et al. [43]	2021	Russia	Cross sectional	57	1358	49.70%	15.5(211)
G. Nesan et al. [44]	2021	India	Cross sectional	≤ 10-≥60	1354	73%	39.7(538)
G.F. Parisi et al. [45]	2021	Italy	Cross sectional	20->60	267	34.4%	75.6(202)
M. S. Razai et al. [47]	2021	London	Cross sectional	49	41	34%	45(19)
E. Righi et al. [47]	2021	Italy	Cohort	56	417	N/A	11.27(47)
L. Roge et al. [48]	2021	Latvia	Cross sectional	10	236	56%	25.2(59)
J. Seeble et al. [49]	2021	Germany	Cohort	57	96	44.8	53.1(51)
S. Sultana et al. [50]	2021	Bangladesh	Cross sectional	34.8	186	66%	8.1(15)
M. Taquet et al. [51]	2021	UK	Cohort	39.4	106,578	41.6%	6.38(6799)
R. R. taylor et al. [52]	2021	UK	Cross sectional	N/A	675	58%	54.5(368)
R. Titze-de-Almeida et al. [53]	2022	Brazil	Cohort	41.2	236	39%	21.2(50)
B. S. Tomar et al. [54]	2021	India	Cross sectional	53.4	50	76%	74(37)
G. Vanichkachorn et al. [55]	2021	USA	Cohort	45.4	100	32%	80(80)
V. Wanga et al. [56]	2021	USA	Case control	39.9	698	52%	48.4(338)
F. Bai et al. [57]	2021	Italy	Cohort	57	377	63.7%	15.6(59)
S. Naik et al. [58]	2021	India	Cross sectional	41.6	1234	69.4%	5.5(68)
N. Yaksi et al. [59]	2022	Turkey	Cohort	65.7	133	51.8%	45.9(61)
L. Simani et al. [60]	2021	Iran	Cross sectional	54.62	120	66.7%	17.5(21)
M. Van Herck et al. [61]	2021	Netherlands	Cross sectional	50	239	17.2%	85.4(204)
J. Bungenberg et al. [62]	2022	Germany	Cross sectional	50.5	50	44%	66(33)
F. Zhou et al. [63]	2020	China	Cohort	56	191	62%	23(44)
S. Mandal et al. [64]	2020	London	Cross sectional	59.9	384	62%	69(265)
C. Fernandez-de-las-Penas et al. [65]	2021	Spain	Cohort	61	1142	52.5%	61(695)
Q. Xiong et al. [66]	2020	China	cross-sectional cohort	20–80	538	45.5%	28.6(144)
KB. Jacobson et al. [67]	2021	USA	Cross sectional	43.3	118	53.4%	30.8(36)
W. Shendy et al. [68]	2021	Egypt	Cross sectional	34.03	81	32%	64.2(52)
J. A. Gonzalez-Hermosillo et al. [69]	2021	Mexico	Cohort	51	130	65.4%	53(69)
S. J. Halpin et al. [70]	2020	UK	Cross sectional	70.5	68	51.5%	14.7(10)
D. Liu et al. [71]	2020	Netherlands	Cross sectional	43	149	45%	26.2(38)
L. Tabacof [72]	2020	USA	Cross sectional	44	84	31%	92(77)
L. G. Jacobs et al. [73]	2020	USA	Cross sectional	57	183	61.5%	83.2(149)
S. Zou et al. [74]	2020	China	Cross sectional	62.8	1063	32.6%	47.1(501)

**Fig. 2:**
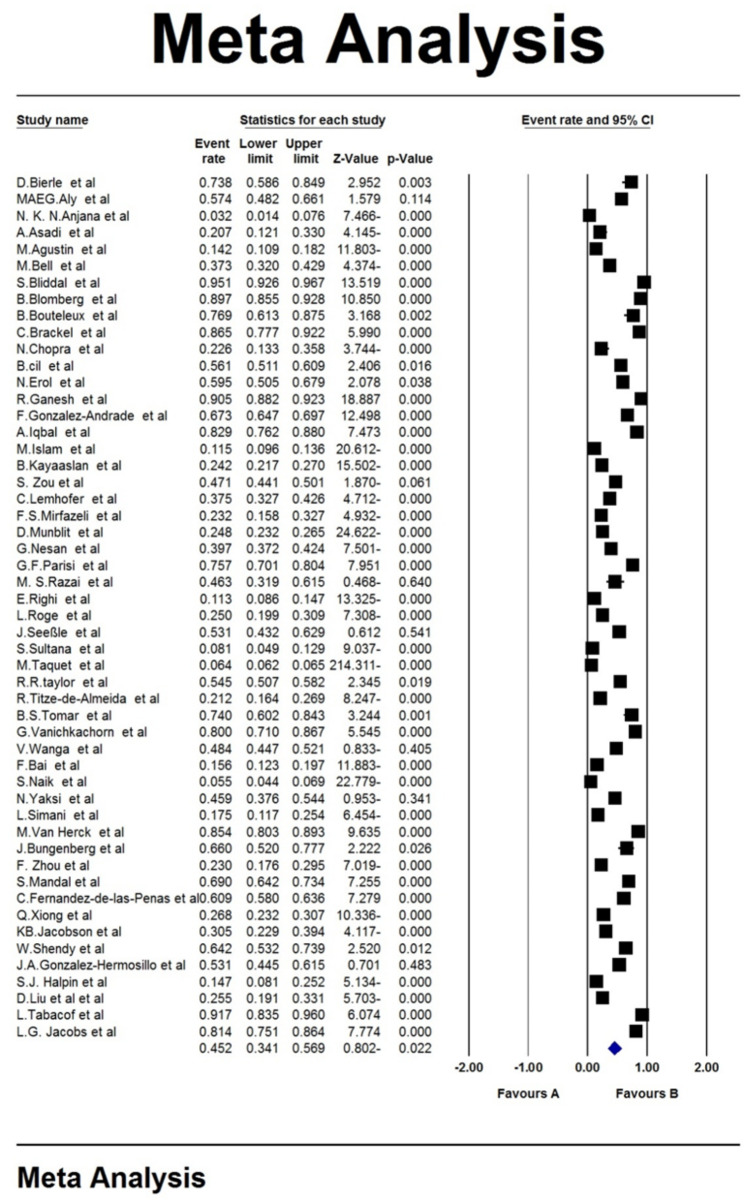
Forest plot of pooled global prevalence of chronic fatigue syndrome among long COVID patients based on a random effects model

**Fig. 3:**
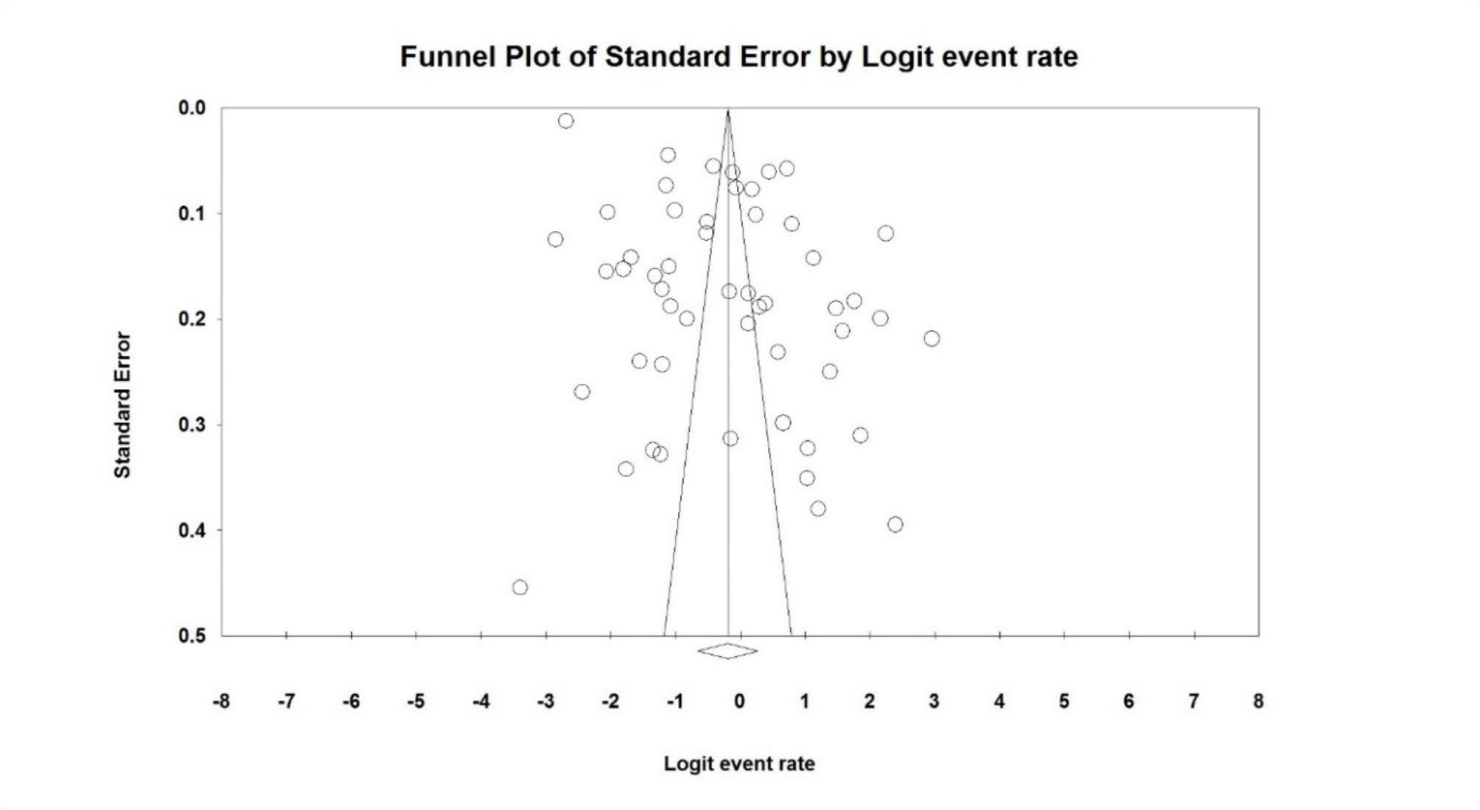
Funnel plot on publication bias results among studies on global prevalence of chronic fatigue syndrome among long COVID patients

To evaluate the heterogeneity of studies with the I^2^ test (I^2^: 99.7), due to the high heterogeneity in the studied studies, a random effects model was used in the meta-analysis of results. The pooled global prevalence of chronic fatigue syndrome among long COVID patients was found to be 45.2% (95% CI: 34.1-56.9%) (Fig.2). The study of publication bias in the studies were examined with the Begg’s test (due to the high sample size in the studies), at a significance level of 0.1, and publication bias was found to be statistically significant (p = 0.007) Therefore, considering the significance of the publication bias, the reported results should be interpreted with caution (Fig.3).

**Fig. 4:**
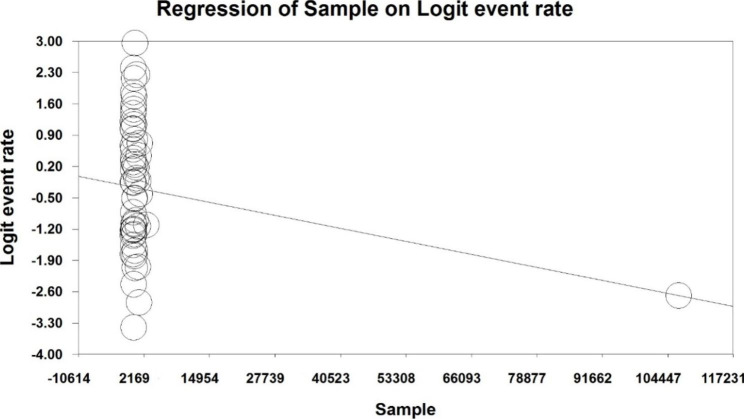
Meta-regression prevalence of chronic fatigue syndrome among long COVID patients, in terms of sample size

**Fig. 5:**
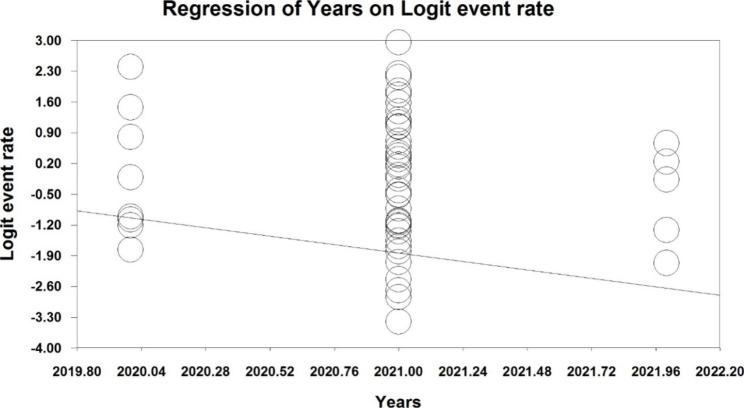
Meta-regression of prevalence of chronic fatigue syndrome among long COVID patients, by year

Due to the high heterogeneity reported in the studies, meta-regression test was used to investigate the effect of two factors: the sample size, and the year of study (p < 0.05). Meta-regression analysis in examining the effects of the two factors of sample size, and year of study on the changes in the overall prevalence, showed that with increasing sample size, and year of study, the prevalence of chronic fatigue syndrome among long COVID patients (Fig.4, and 5).

## Discussion

The present study is the first systematic review and meta-analysis on the global prevalence of chronic fatigue syndrome among long COVID patients. This study has been compiled using the most optimal secondary analysis methods from among 52 eligible studies. The total study population was 127,117 and the pooled global prevalence of chronic fatigue syndrome among long COVID patients was found to be 45.2% (95% CI: 34.1-56.9%).

According to several studies, it has become apparent that many COVID-19 patients may have persistent symptoms even after acute infection has been treated. These symptoms may be specific to COVID-19 or secondary symptoms related to long-term hospitalization, including hospitalization in intensive care units. The most common symptoms of long COVID are physical and psychological symptoms [57, 75]. Symptoms reported among long COVID-19 patients include fatigue, musculoskeletal disorders, sleep problems, headache, cognitive problems, sore throat, lethargy, vertigo, and palpitations after four weeks from the onset of the disease [24]. Fatigue and shortness of breath were the predominant symptoms, which have been reported for a long time and the most common symptom reported among long COVID cases is “chronic fatigue” [25].

The prevalence of fatigue has been indicated in many studies reviewed in this systematic review, as the most common symptom among patients with post-COVID-19 syndrome [25, 28, 34, 36, 37, 40, 41]. What causes long COVID-19 symptoms, including chronic fatigue, and why do some people experience them are still under further exploration. However, there are a number of causes suggested in existing studies including virus dose and host dependence or host resistance [49]. Low-grade muscle damage and inflammation, neuropathy, myopathy following COVID-19, and immobility due to the severity of the disease may cause fatigue [52, 54]. Additionally, lung disorders caused by COVID-19 are suggested as other triggers [76].

Studies show that despite receiving medical and health care, patients with severe fatigue may show little or no improvement 3 to 6 months after seeing this symptom, and chronic long COVID fatigue may persist for more than six months [61]. There is a fact that a number of patients may experience distressing fatigue which can have major effects on the individual’s life, the health system and the wider community [77–79]. Instances of such negative impacts include health care costs, sick leave, and increased consumption of medical care [82] and decreased quality of life among patients [61, 83, 84].

In a Swedish study of healthcare professionals, 8% of participants reported disturbances in their work life, and 15% reported disturbances in their social life [9]. The findings show that patients with post-COVID-19 syndrome not only suffer from the symptoms, but also suffer more due to their reduced ability to participate in individual and social activities. Consequently, their day to day lives are disrupted [36]. In one study, 49% of patients experienced long COVID symptoms and chronic fatigue. Social disability has also been reported to be more than 30% participants in the study by Lemhofer [41]. Among children, it was also reported that affected children had experience disruptions in their social activities due to post-COVID fatigue and other post-COVID symptoms. For instance, in a study of a population of children, none of the children aged 6 to 8 in this study, have been able to continue going to school [85]. Research have shown that one of the reasons for the decrease in the ability of people in participation in social activities in all age groups can be the fact that after recovery, they often experience discrimination and prejudice due to society’s irrational fear contagiousness of recovered individuals; this further reduces social relations and social activities [38].

Studies show that the prevalence of post-COVID fatigue is higher among women than men [49]. Similarly, a study conducted in the United States and the United Kingdom shows that the prevalence of fatigue among women is three times higher than men; This may be because of hormones that cause the acute inflammatory phase to continue even after the infection has treated [57]. It has also been suggested that in addition to the high prevalence of fatigue among women, this symptom is more persistent in women than men, which may be due to the stronger level of IgG antibody, which helps to improve symptoms, yet if the presence of this high-level antibody in the body continues, it will cause physical manifestations [29, 36]. It is also argued in literature that the higher prevalence of post-COVID symptoms among women is due to the fact that many women are working in the healthcare systems which causes more contact with COVID-19 patients [29]. Moreover, it was argued in a study that this higher prevalence may be due to women paying more attention to their own well-being and bodies, who are more aware of changes and distresses [57]. However, some studies have not reported differences in the prevalence of fatigue between men and women [86–88].

Regarding the prevalence of chronic post-COVID fatigue in different age groups, studies in the early stages of the pandemic stated reported that there were no complications due to COVID-19 among children. However, further research conducted specifically on children showed that children with COVID-19 do not necessarily get or demonstrate a weaker COVID-10 compared to adults, and also, they have no greater protection against long-term complications of COVID-19 [45]. In one study, chronic post-COVID fatigue was reported to be the predominant symptom experienced in all age groups with post-COVID-19 syndrome [48]. In the study by Asadi-Pooya, the prevalence of chronic post-COVID-19 fatigue was 69% in the adult population, whereas in study of Mandal 7% in children and adolescents reported post-COVID-19 fatigue [26, 64]. In a study among the youth population (30–30 years old), chronic COVID-19 fatigue was reported to be 21% [30]. Following these results, a study conducted in Ireland confirms that aging is significantly associated with fatigue [80]. In general, most studies have confirmed the relationship between fatigue and age, and it has been stated that the prevalence and severity of fatigue among the adult group is higher than the lower age groups [47], which can be due to the link between fatigue and hospitalization that is more prevalent among older patients [47].

In a study by Bungenberg et al., the prevalence and severity of COVID-19 fatigue were higher among non-hospitalized patients [62]. Moreover, in the study of Çil et al., hospitalized patients suffered with more post-COVID-19 fatigue than non-hospitalized individuals [34]. It is also reported that the length of hospital stay affects the duration of chronic post-COVID-19 fatigue [47]. A review article found that the prevalence of post-COVID-19 symptoms among hospitalized patients was twice as high as in outpatients [59]. A study of work leaves durations among patients that were hospitalized was more than a month longer than non-hospitalized patients, which may indicate a greater severity of chronic post-COVID-19 fatigue among hospitalized individuals [89]. Although hospitalization is observed in all age groups, the hospitalization rate is higher among adult age groups. Accordingly, the greater severity of post-COVID 19 symptoms among the adult age group, could be considered as one of the reasons for the high severity of chronic post-COVID-19 fatigue in hospitalized patients [59]. Additionally, because people with more severe illness due to COVID-19 are hospitalized and their hospital stay is longer, and since the prevalence of post-COVID 19 symptoms is higher among patients with more severe illness, it can be concluded that there is a relationship between hospitalization and the prevalence of fatigue [59]. According to a study by Bungenberg et al., it can be concluded that a number of post-COVID-19 symptoms, including fatigue are caused due to ICU treatments and hospitalization [62]. In other words, post-COVID-19 symptoms including fatigue are the result of long-term hospitalization and follow-up intensive care [57].

## Limitations

Our study has its limitations. All of the analysed studies were cross-sectional, cohort, and case studies that examined individuals’ status only over a limited period of time. We also reviewed studies provided that at least four weeks had elapsed since the onset of symptoms, which may have less persistent symptoms in some individuals. There is a lack of a more accurate definition of chronic fatigue and a specific criterion for measuring it would also be helpful. Future studies with clearer follow-up of patients and more detailed examination of symptoms through more accurate physical assessments and medical tests, and more specific criteria on chronic fatigue can provide clearer results. Although according to the entry and exit conditions in our study, we tried to search all available databases based on relevant keywords, but one of the most obvious limitations of the present study is the significance of the publication bias, which undermines the main results of the study and shows It provides unpublished studies that we could not find.

## Conclusion

Our findings show that the pooled global prevalence of chronic post-COVID-19 syndrome is 45.2%. The prevalence of chronic post-COVID-19 fatigue affects people’s individual and social lives materially and spiritually. Moreover, the results of some studies indicate that this prevalence is higher among women, and adults. Given the challenges and negative impacts that this syndrome causes to individuals, it is recommended that more studies be conducted to derive further insights, and it is also recommended that health policymakers allocate further funds to reduce the adverse effects of this syndrome, with such support could include creating a pertinent support program.

## Data Availability

Datasets are available through the corresponding author upon reasonable request.
